# Nicotine receptors mediating sensorimotor gating and its enhancement by systemic nicotine

**DOI:** 10.3389/fnbeh.2015.00030

**Published:** 2015-02-11

**Authors:** Farena Pinnock, Daniel Bosch, Tyler Brown, Nadine Simons, John R. Yeomans, Cleusa DeOliveira, Susanne Schmid

**Affiliations:** ^1^Anatomy and Cell Biology, Schulich School of Medicine and Dentistry, University of Western OntarioLondon, ON, Canada; ^2^Department of Psychology, University of TorontoToronto, ON, Canada; ^3^Hertie Institute for Clinical Brain Research, Eberhard-Karls-UniversitätTübingen, Germany

**Keywords:** prepulse inhibition, sensory gating, sensorimotor gating, startle, rat, schizophrenia, nicotine receptor, alpha-7 nicotine receptor

## Abstract

Prepulse inhibition (PPI) of startle occurs when intensity stimuli precede stronger startle-inducing stimuli by 10–1000 ms. PPI deficits are found in individuals with schizophrenia and other psychiatric disorders, and they correlate with other cognitive impairments. Animal research and clinical studies have demonstrated that both PPI and cognitive function can be enhanced by nicotine. PPI has been shown to be mediated, at least in part, by mesopontine cholinergic neurons that project to pontine startle neurons and activate muscarinic and potentially nicotine receptors (nAChRs). The subtypes and anatomical location of nAChRs involved in mediating and modulating PPI remain unresolved. We tested the hypothesis that nAChRs that are expressed by pontine startle neurons contribute to PPI. We also explored whether or not these pontine receptors are responsible for the nicotine enhancement of PPI. While systemic administration of nAChR antagonists had limited effects on PPI, PnC microinfusions of the non-α7nAChR preferring antagonist TMPH, but not of the α7nAChR antagonist MLA, into the PnC significantly reduced PPI. Electrophysiological recordings from startle-mediating PnC neurons confirmed that nicotine affects excitability of PnC neurons, which could be antagonized by TMPH, but not by MLA, indicating the expression of non-α7nAChR. In contrast, systemic nicotine enhancement of PPI was only reversed by systemic MLA and not by TMPH or local microinfusions of MLA into the PnC. In summary, our data indicate that non-α7nAChRs in the PnC contribute to PPI at stimulus intervals of 100 ms or less, whereas activation of α7nAChRs in other brain areas is responsible for the systemic nicotine enhancement of PPI. This is important knowledge for the correct interpretation of behavioral, preclinical, and clinical data as well as for developing drugs for the amelioration of PPI deficits and the enhancement of cognitive function.

## Introduction

Prepulse inhibition (PPI) describes the attenuation of a reflexive motor response due to the presentation of a sensory stimulus prior to the response eliciting stimulus, and can be easily measured in humans and animals using the acoustic startle response. Sensory processing of e.g., the prepulse is facilitated when colliculus-mediated orienting responses occur, leading to inhibition of the startle pathway at the same time. In particular, mesopontine cholinergic arousal systems provide diffuse thalamocortical activation while inhibiting startle (Fendt et al., 2001; Yeomans, 2012). PPI disruptions are a hallmark of schizophrenia, but PPI is also disrupted in several other neurological disorders (for review see Powell et al., 2012; Kohl et al., 2013; Swerdlow, 2013), and PPI disruptions are correlated with other cognitive dysfunctions, such as disorganized thought, attention deficits and working memory disruptions (Braff et al., 1978; Geyer and Braff, 1987; Young et al., 2009; Singer et al., 2013).

The mesopontine cholinergic projection from the pedunculopontine and laterodorsal tegmentum (PPT/LDT) to the startle mediating giant neurons in the caudal pontine reticular nucleus (PnC) has been identified as a crucial system for inhibiting startle during PPI (for review see Koch, 1999; Fendt et al., 2001). Lesions to the PPT/LTD severely attenuate PPI in rats (Kodsi and Swerdlow, 1997; Jones and Shannon, 2004; but see MacLaren et al., 2014) and stimulation of this projection activates muscarinic receptors inhibiting startle-mediating giant neurons in the PnC (Bosch and Schmid, 2006). In addition, there is evidence that ionotropic nicotine receptors (nAChRs) contribute to this cholinergic effect (Bosch and Schmid, 2008). In fact, the activation of both ionotropic and metabotropic receptors have been suggested to mediate the fast (10–100 ms) and long-lasting (100–1000 ms) effects of PPI, respectively (Jones and Shannon, 2000a; Yeomans et al., 2010). Thus, pontine nAChRs might play a role in mediating PPI especially at shorter intervals between prepulses and startle stimuli.

Systemic administration of nicotine has also been shown to enhance PPI in healthy human participants (Warburton, 1992; Warburton et al., 1992; Kumari et al., 1996; Della Casa et al., 1998; Levin et al., 1998b; Mancuso et al., 1999; Levin and Rezvani, 2006), in individuals with schizophrenia (Kumari et al., 2001; Postma et al., 2006; Hong et al., 2008), and in rodents (Acri et al., 1994; Curzon et al., 1994; Levin et al., 1998a; Faraday et al., 1999; Azzopardi et al., 2013). It is not known if nicotine affects PPI directly within the PnC, or indirectly by modulating neuronal activity in higher brain regions that exert modulatory control over the startle and PPI circuitry, such as the hippocampus, striatum, medial prefrontal cortex, or amygdala (for review see Koch, 1999).

In this study we tested the hypothesis that the activation of nAChRs located in the PnC contributes to PPI, particularly at short interstimulus intervals, using systemic and local PnC microinfusions of nAChR antagonists and agonists in rodents. Subsequently, following the identification of the nAChR subtype(s) involved, using behavioral and electrophysiological approaches, we tested whether or not the same nAChRs are responsible for the systemic nicotine enhancement of PPI.

## Materials and methods

### Behavioral experiments

#### Animals

Male adult Sprague Dawley rats, weighing 250–350 g, were obtained from Charles River Canada (St. Constant, QC, Canada). Adult rats were housed in groups of two or three (except during recovery period after surgery) at a temperature of 21 ± 1°C in individual cages on a 12 h light/dark cycle with lights on at 7 a.m., and food and water available *ad libitum*. All animal procedures were approved by the University of Western Ontario Animal Care Committee and followed the guidelines of the Canadian Council on Animal Care. All efforts were made to minimize the number of animals used and any discomfort resulting from surgical or behavioral procedures. Testing occurred during the light part of the light/dark cycle to minimize movement during testing in these nocturnal animals.

#### Surgery

For local microinfusions into the PnC, rats were anaesthetized, using a mixture of 2% isoflurane and 98% oxygen. A subcutaneous injection of 0.05 mg/kg of buprenorphine and 5 mg/kg of ketoprofen was given during surgery for analgesia. Blunt-ended ear bars and a snout mask were used to secure the head in the stereotaxic device. Two 23-gauge stainless steel guide cannulae (Plastics One, Roanoke, VA, USA) were stereotaxically aimed bilaterally at the ventrocaudal segment of the PnC based on coordinates derived from the Paxinos and Watson rat brain atlas (Paxinos and Watson, 2005). Coordinates relative to Lambda were: +2.5 mm in the medial/lateral plane; −8.80 mm in the ventral/dorsal plane; −2.1 mm in the rostral/caudal plane, with a 10 degree mediolateral angle. The cannulae were mounted on the skull with dental cement casted around four jeweler's screws implanted bilaterally into the parietal skull plates. Stainless steel stylets were inserted into cannula to prevent clogging (PlasticsOne, Roanoke, VA, USA). Silk suture was used to close the wound, and rats were allowed a 7-day recovery period in the animal care facility.

#### Handling

Approximately 3 days before testing, rats were handled to ensure familiarity with the handler and the startle boxes. Rats were socialized for approximately 10 min each day for 3 days. On the second and third handling days, rats were afterwards placed in the startle apparatus for 5–10 min while the constant sound of a 65 dB white noise played in the background.

#### Injections/microinfusions

Each rat received two systemic injections or local microinfusions unless otherwise noted: one drug and one vehicle, in a pseudo-randomized order and at least 5 days apart. Stereotaxic microinfusions were accomplished with 30 G Infusion cannulae that were inserted into the guide cannulae and extended 1 mm beyond the tip of the guide cannulae. 0.5 μ L of drug or vehicle was injected bilaterally in awake rats over a 4-min period, using a mechanical syringe pump (World Precision Instruments, Sarasota, FL). Infusion cannulae remained inside the guide cannulae for an extra minute to ensure complete diffusion of drug.

#### Startle testing

After injections or microinfusions, rats went back to their home cage and were placed in startle chambers (Med Associates, Vermont, USA) 5–20 min after injection, unless otherwise noted. The startle reflex software SOF-825, version 5.95 (Med Associates, Vermont, USA) was used to perform experiments and analyze data. Rats were first subjected to a 5 min acclimation period with a 65 dB white noise background without further stimulation. They were then exposed to 30 pulse-alone trials (Block 1) for habituation. Immediately following Block 1, they were subjected to up to 60 pseudo-randomized trials consisting of different stimulation trials, each administered 10 times (Block 2): a pulse-alone trial, and up to five different prepulse-pulse trials with the following Interstimulus intervals (ISI) between prepulse and startle pulses: 12, 20, 50, 100, and/or 250 ms. The startle evoking pulse always consisted of a 20 ms long burst of white noise of 105 dB. Two different prepulse intensities, either 75 or 85 dB, were used as indicated. All prepulses consisted of a 4 ms long burst of white noise. Trials were 20 s apart. For a more detailed description, see (Valsamis and Schmid, 2011). Protocols were shortened by omitting trial types in experiments involving injections of nicotine, since nicotine has a short half-life.

#### Histology

Rats that had received microinfusion through cannula implants were injected with a lethal dose of pentobarbital and received a microinfusion of a small amount of 3% thionine dye through the implanted cannulae in order to mark placements. Rats were then perfused transcardially. Brains were harvested and post- fixed by immersion in 4% paraformaldehyde (PFA) for at least 1 h, and then transferred to 15% sucrose (in buffer) for another 24 h. Brains were sliced into 50 μm-thick sections by a freezing microtome. Sections were mounted, dried, and stained using the Haematoxylin and Eosin counterstaining procedure. Cannulae coordinate determination was made using a rat brain atlas by Paxinos and Watson (2005). Microinfusion tips that reached or penetrated the borders of the PnC were deemed as successful hits. All other placements were deemed as misses and their data were discarded.

#### Data analysis

The 10 startle responses that were preceded by a specific prepulse were averaged and divided by the average startle responses that had no preceding prepulse in block 2. These numbers were then subtracted from 1 and multiplied by 100 to yield a “Percent PPI” score:

$$\begin{array}{l} \text{\% }prepulse\;inhibition\text{ = }\\ \;\;\;\;\;\;\;\;\;\;\left(1-\frac{\left(prepulse-pulse\;trial\;amplitude\right)}{\left(pulse-alone\;trial\;amplitude\right)}\right)*100 \end{array}$$

Averages of both PPI intensities were taken for every ISI, and Two-Way, repeated measures ANOVAs were conducted using GB Stat software (GB Stat®). If warranted, a Fischer's Least Significance Difference (LSD) *post-hoc* test was used to assess points of significance. Baseline startle measurements were calculated by averaging, for each rat, the first 30 startle alone trial responses in block 1. These individual baseline startle scores were analyzed for different drug conditions using a two-tailed, paired Student's *t*-test. In both the ANOVAs and the Student's *t*-tests, differences in the data were deemed significant if *p*-values were less than 0.05 (α = 0.05).

### Electrophysiology

#### Slice preparation

A more detailed description of the slice preparation containing the PnC giant neurons and their afferent projections within the startle pathway has been published previously (Weber et al., 2002; Simons-Weidenmaier et al., 2006; Schmid et al., 2010, see also Supplementary Figures S1, S2). In brief, juvenile Sprague-Dawley rats (P9-14, with P1 defined as the day of birth) were anaesthetized with isoflurane and their brains rapidly removed and transferred into ice-cold preparation solution containing (in mM): KCl, 2; MgCl_2_, 2; KH_2_PO_4_, 1.2; MgSO_4_, 1.3; NaHCO_3_, 26; glucose, 10; saccharose, 210; CaCl_2_, 2; myoinositol, 3; sodium pyruvate, 2; ascorbic acid, 0.4; equilibrated with 95% O2/5% CO_2_. Coronal slices of 350–400 μm thickness were cut with a vibratome (Microm, Walldorf, Germany) in a submerged chamber filled with ice-cold preparation solution and transferred into a holding chamber filled with artificial cerebrospinal fluid (ACSF) containing (in mM): KCl, 2; KH_2_PO_4_, 1.2; MgSO_4_, 1.3; NaHCO_3_, 26; NaCl, 124; glucose, 10. CaCl_2_ (2 mM) was added a few minutes after slices had been transferred. In order to improve later patch success the holding chamber was heated for 30 min to 32–35°C. Slices were then kept at room temperature for at least an additional 30 min.

#### Electrophysiological recordings

For recording, slices were transferred into a superfusion recording chamber mounted on an upright microscope (Zeiss, Oberkochen, Germany) with an infrared sensitive camera (Kappa, Germany). Superfusion rate was 2–3 ml ACSF/min at room temperature and patch-clamp recordings have been carried out under visual guidance. Patch electrodes were pulled out of borosilicate capillaries (Science Products, Hofheim, Germany) and filled with a solution containing (in mM): K-gluconate, 130; EGTA, 0.5; MgCl_2_, 2; KCl, 5; HEPES, 10; pH 7.2 (KOH), 270–290 mosm. Electrodes had a resistance of 2.3–3.5 MΩ. The calculated junction potential using pClamp 10 is 11.8 mV and data was not corrected for it.

Giant neurons in the PnC were identified by a soma diameter greater than 35 μm and all recordings were made in voltage-clamp mode at a holding potential of −70 mV unless otherwise noted. Resting membrane potentials were measured by briefly switching into current clamp mode (*I* = 0). Presynaptic stimuli were applied by bipolar tungsten electrodes (Science Products). One stimulation electrode was positioned medial to the principal sensory trigeminal nucleus (Pr5) and the seventh nerve in order to stimulate trigeminal afferents. The second electrode was positioned ventral to the lateral superior olive for auditory afferent fiber stimulation. Both electrodes were connected via isolators to a pulse generator (Master-8, Science Products). Stimulus pulse duration was always 150 μs. Stimulus intensities were kept low to avoid spiking of the postsynaptic neurons. Recordings were made using an Axopatch 200B amplifier and digitized by Digidata 1200 (both Axon Instruments, Union City, USA). The data was filtered with a 5 kHz low-pass filter, the sampling rate was 20 kHz. The pClamp 8.2.0 software (Axon Instruments) was used for data acquisition and analysis. Series resistance and seal quality were monitored at the beginning and several times throughout the recordings. Only one cell was recorded per slice, and there was only one slice per rat in most cases. Statistical analysis was performed using SPSS. When only one drug/dose was tested, a Student's *t*-test was perfomed, and the *p*-value is reported. When several drugs or doses were tested, a repeated measurement mixed design ANOVA was used and both *F*- and *p*-values are reported. A LSD test was used for *post-hoc* analysis. In both the ANOVAs and the Student's *t*-tests, differences in the data were deemed significant if *p*-values were less than 0.05 (α = 0.05).

### Drugs

The following drugs were used in behavioral and electrophysiological experiment in concentrations as indicated in the Results section: Liquid (−)-Nicotine (Sigma Chemical Co. Ltd., USA, concentration reflects the free base), the cholinergic receptor agonist carbachol (Sigma Chemical Co. Ltd., USA), the natural alkaloid and nicotine receptor blocker (+)-tubocurarine chloride (Tocris Bioscience, USA), the natural alkaloid and specific alpha-7 nicotine receptor blocker methyllycaconitine (MLA; Tocris Bioscience, USA), the highly alpha-7 specific agonist N-[(3R)-1-azabicyclo[2.2.2]oct-3-yl]furo[2,3-c]pyridine-5-carboxamide hydrochloride (PHA 543 613; Tocris Bioscience, USA) and the non-α7 preferring non-competitive nAChR antagonist, 2,2,6,6-tetramethylpiperidin-4-yl heptanoate (TMPH) hydrochloride (Tocris Bioscience, USA). Drugs were dissolved in double distilled water in a 100X stock solution and kept at −18°C until used in behavioral experiments. Before usage, they were diluted in physiological saline. Drug concentrations were selected based on previously published studies (nicotine: Hamann and Martin, 1992; Acri et al., 1994; Schreiber et al., 2002; MLA: Panagis et al., 2000; Chilton et al., 2004; TMPH: Damaj et al., 2005). For electrophysiological experiments, drugs were dissolved in a 1000^*^ stock solution in double distilled water and they were added to the bath solution (1:1000) during experiments. Cadmium (100 μM; Sigma-Aldrich, Canada) was prepared as a 50 mM CdCl_2_ stock solution and added to the perfusing (oxygenated) ACSF to block synaptic transmission by blocking voltage-gated calcium channels.

## Results

### Systemic nicotine antagonists

**Figure 1:**
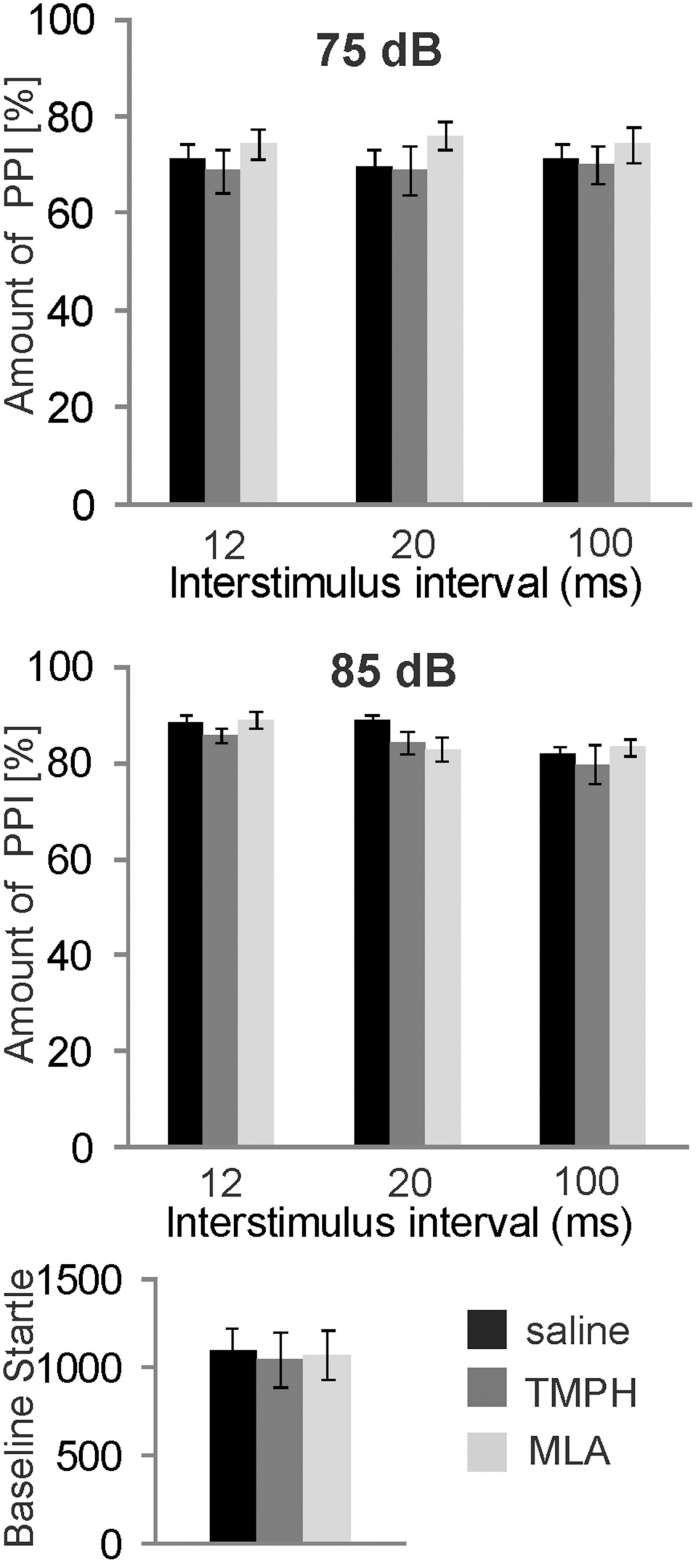
**Systemically injected nicotine antagonists do not affect PPI**. Prepulse inhibition was measured using a 75 or 85 dB white noise prepulse preceding the startle pulse by different intervals, as indicated. The amount of PPI was calculated using the intermingled trials without prepulse as baseline startle (0% PPI). Rats were systemically injected with saline, the α7nAChR preferring antagonist MLA, or the non-α7nAChR antagonist TMPH at doses previously shown to be effective. There was no effect of drug on PPI at prepulse levels of 75 dB (*top*), or 85 dB *(middle*). Baseline startle amplitudes were analyzed during the habituation block (see Materials and Methods; *n* = 8 rats per group).There was also no effect of drug on the baseline startle response amplitude (*bottom*).

In order to test the role of nAChRs in prepulse inhibition, we systemically injected a group of 8 rats with the non-α7nAChR preferring antagonist TMPH and the α7nAChR antagonist MLA, at a dose of 10 mg/kg which has been shown by others to be highly effective (Damaj et al., 2005; Gao et al., 2010). Each rat received both drugs and a vehicle injection in a pseudorandomized order, and at least 5 days apart, for within subject comparison. Systemic administration of nAChR antagonists did not alter PPI [*F*_(2, 34)_ = 1.896; *p* = 0.200; Figure 1, *top*]. There was also no effect on baseline startle amplitudes [*F*_(2, 34)_ = 0.108; *p* = 0.200; Figure 1, *bottom*].

### Nicotine and nAChR antagonists in the PnC

**Figure 2:**
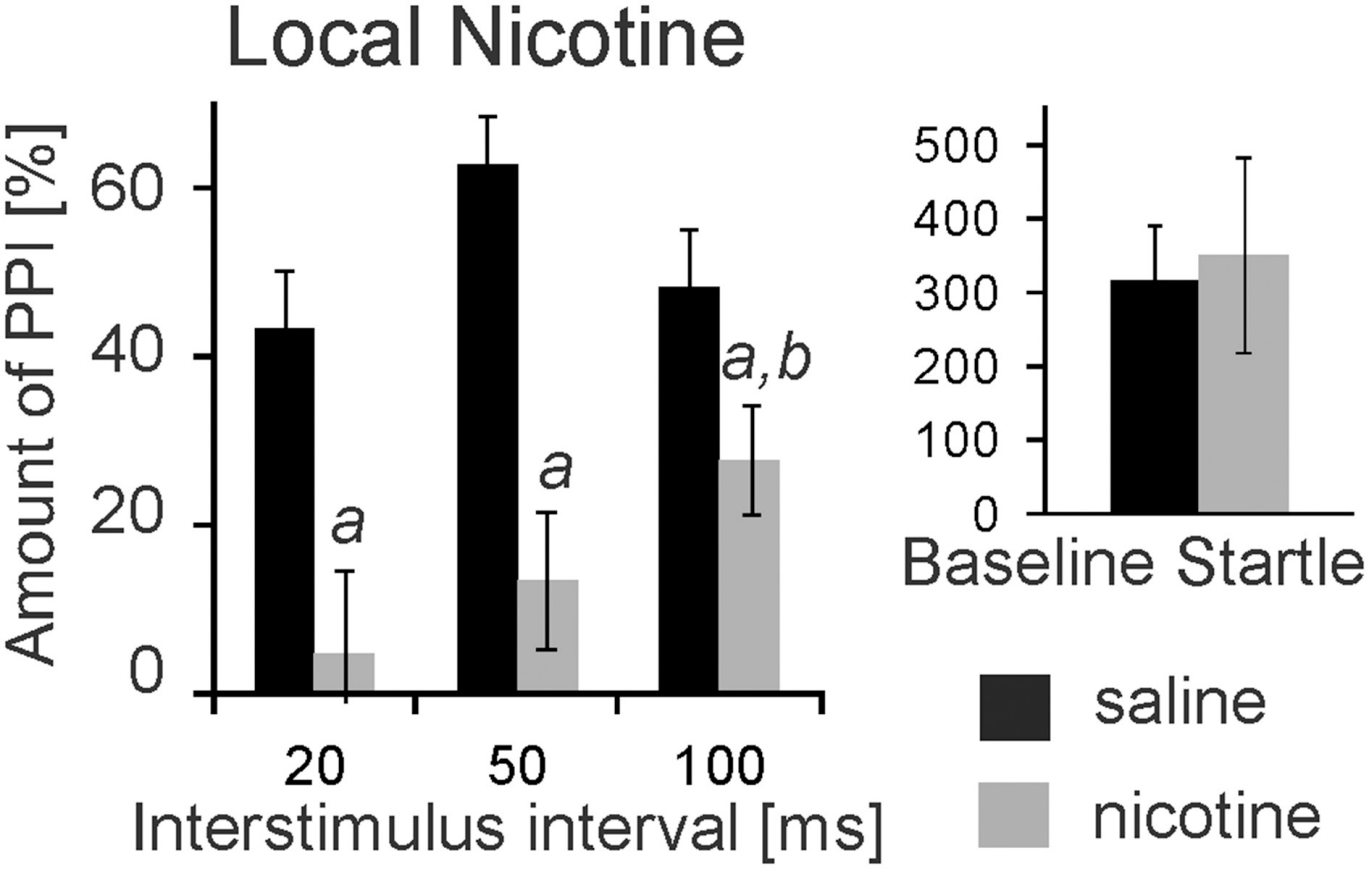
**Local PnC microinfusions of 0.5 μl nicotine (10 mM) impair PPI**. Rats were bilaterally injected with 10 mM nicotine (in 0.5 μl saline) or vehicle through chronically implanted cannulae targeting the PnC. They were immediately tested for startle and PPI using a 75 db white noise prepulse. The amount of PPI was significantly decreased in rats injected with nicotine when compared to rats injected with saline (labeled by “*a*”). The decrease was significantly stronger at 20 ms interstimulus intervals compared with 100 ms intervals (labeled by “*b*”). Baseline startle amplitudes did not significantly differ between groups (*n* = 9 rats).

nAChRs are abundantly expressed and might mediate opposing effects on PPI when targeted systemically. We therefore, targeted nAChRs directly in the PnC where the mesopontine cholinergic neurons supposedly synapse on startle mediating giant neurons, using microinfusions through chronically implanted bilateral cannulas prior to behavioral testing. Post-mortem histology confirmed PnC placement of cannulas for a group of 9 rats. Data of 3 rats were discarded since one or both cannulas missed the PnC. Intracranial (i.c.) microinfusions of 10 mM nicotine (0.5 μl) revealed a main group effect of nicotine in these 9 rats [*F*_(1, 17)_ = 67.06; *p* < 0.0001, Figure 2]. A LSD *post-hoc* analysis showed that 5 nmol nicotine severely disrupted PPI at all tested ISIs (20, 50, and 100 ms). Furthermore, the *post-hoc* analysis showed that PPI disruption was significantly stronger at a short ISI of 20 ms compared to a longer ISI of 100 ms. Baseline startle was not affected by microinfusions of nicotine into the PnC (*p* = 0.34, Figure 2).

**Figure 3:**
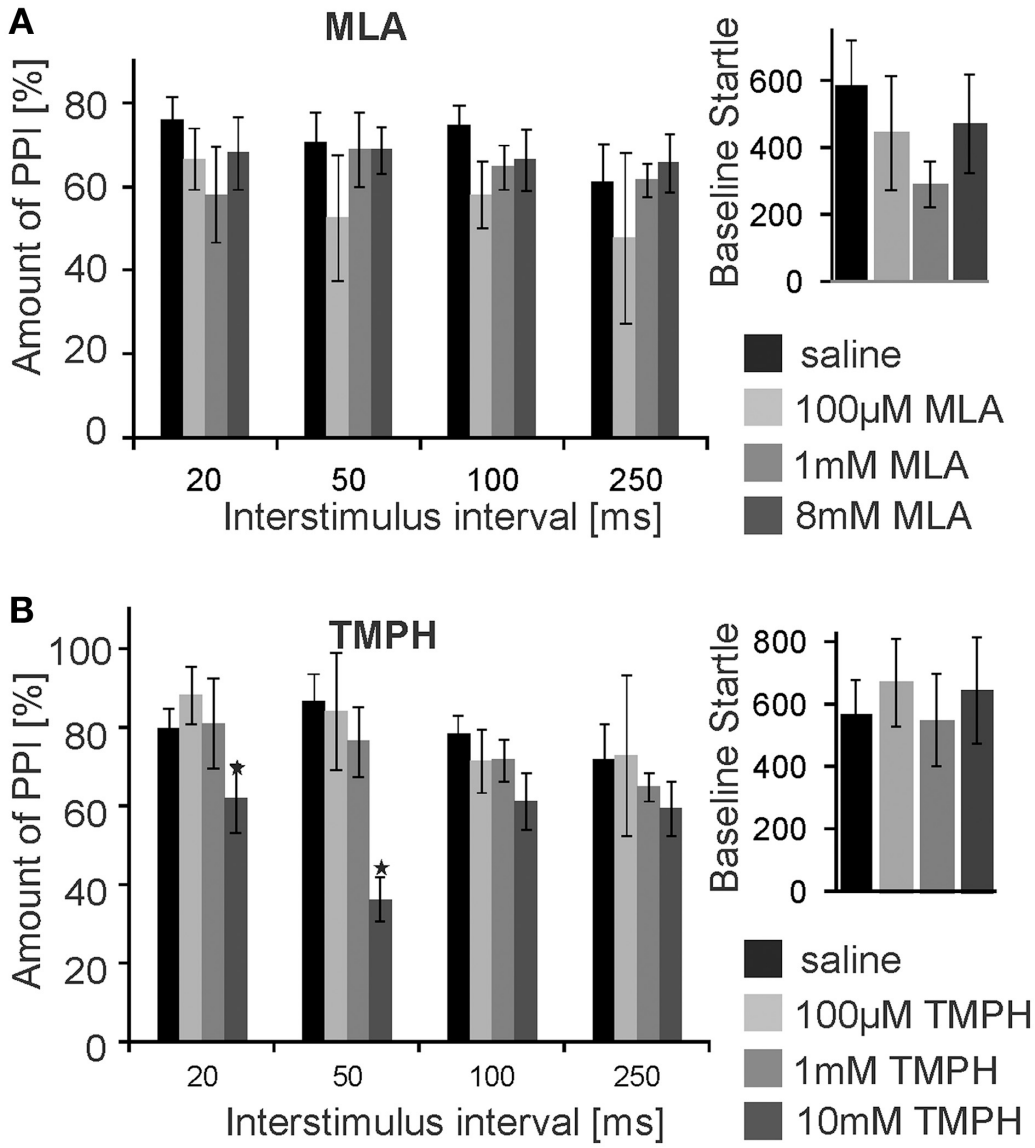
**PnC microinfusion of TMPH, but not of MLA, into the PnC disrupts PPI**. **(A)** Intracranial microinfusions of the α7 preferring nicotinic antagonist MLA into the PnC in doses as indicated does not significantly affect PPI at any ISI. Local MLA infusion has also no significant effect on the baseline startle amplitude (*n* = 6 rats). **(B)** Intracranial microinfusions of TMPH significantly disrupted PPI at the highest dose tested. The effect was restricted to short ISIs of 20 and 50 ms. TMPH did not affect the baseline startle (*n* = 10 rats).

Subsequently, we infused the α7nAChRs antagonist MLA and the non-α7nAChRs preferring antagonist TMPH locally into the PnC. At doses of 0.1, 1, and 8 mM, MLA did not have any significant effect on PPI at any ISI [*F*_(3, 20)_ = 1.90; *p* = 0.15; *n* = 6 animals]. Baseline startle amplitudes were also not affected by PnC microinfusions of MLA (*p* = 0.08; *n* = 6; Figure 3A). In contrast, PnC microinfusions of TMPH hydrochloride at doses of 0.1, 1, and 10 mM showed significant main group effects of drug on PPI [*F*_(3, 36)_ = 115.89; *p* < 0.0001; *n* = 10 animals] with *post-hoc* analysis revealing significant attenuation of PPI for the 10 mM dose of TMPH at short ISIs of 20 and 50 ms, and no significant effect at ISIs of 100 and 250 ms. Baseline startle was not affected by PnC application of TMPH (*p* = 0.11; *n* = 10; Figure 3B). In summary, microinfusions of nicotine antagonists into the PnC indicate a role of non-α7nAChRs in mediating a part of total PPI, specifically at short intervals between prepulse and startle stimuli.

### Effects of nicotine on PnC giant neurons in brain slices

**Figure 4:**
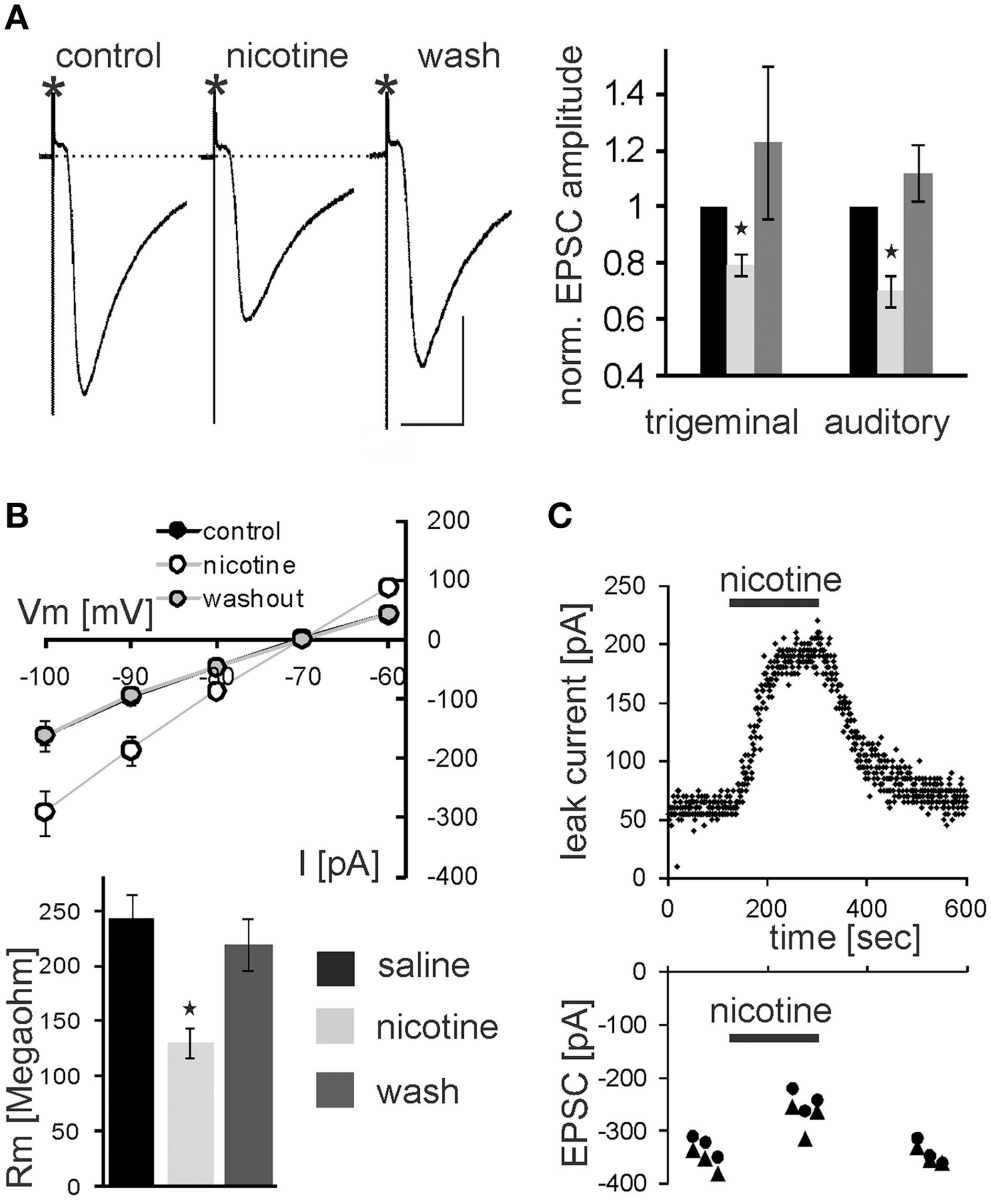
**Nicotine affects synaptic transmission and membrane resistance in PnC giant neurons**. **(A)** Trigeminally evoked excitatory synaptic currents in control conditions, during perfusion with nicotine and after wash out. The asterisks mark the stimulus artifacts. Bars indicate 25 ms (horizontal) and 100 pA (vertical). 10 μM Nicotine significantly reduced EPSC amplitudes following trigeminal and auditory stimulation. This effect was reversed after wash out (*n* = 14 cells). **(B)** Whole-cell membrane leak current amplitudes in response to 10 mV step hyper- and depolarisations from −70 mV to the indicated potentials. Current amplitudes were increased during nicotine perfusion, indicating a lower membrane resistance *(top)*. Calculated membrane resistances (*Rm* = ΔVm/ΔI) show that nicotine significantly reduces membrane resistance in PnC giant neurons *(bottom)*. **(C)** Time course showing whole-cell leak current amplitudes in response to a depolarizing 10 mV voltage step from −70 mV in an exemplary cell before, during, and after nicotine bath application *(top)*, and the corresponding EPSC amplitudes in the same cell *(bottom)*.

In addition to PnC microinfusions of nicotine *in vivo*, we applied nicotine in acute brain slices and monitored glutamatergic synaptic transmission of sensory signaling from afferent startle pathways as well as membrane properties in visually identified startle-mediating PnC giant neurons (see Materials and Methods, Supplementary Figures S1, S2, and Weber et al., 2002). As shown in Figure 4A, bath application of 10 μM nicotine significantly reduced the amplitude of both presynaptically evoked trigeminal and auditory synaptic currents to 79.4 ± 3.5% of control and 72.0 ± 4.2% of control, respectively (*p* < 0.001 and *p* < 0.001, respectively; *n* = 14 cells). After subsequent washout of nicotine EPSC amplitudes recovered to close to control levels (Figure 4C). Paired-pulse ratios with 100 ms IS were unchanged (1.52 ± 0.14 control, and 1.51 ± 0.08 during nicotine). Additionally, membrane resistance was calculated by applying hyper- and depolarizing 10 mV voltage steps and measuring the resulting change in whole cell (leak-) current according to Ohm's law. Membrane resistance of the analyzed PnC giant neurons was significantly reduced during the application of nicotine to 61 ± 5.9% MΩ from an average of 245.14 ± 20.95 MΩ under control condition to an average of 124.92 ± 10.96 MΩ (*p* = 0.002; *n* = 14; Figure 4B). This effect was also reversed by subsequent wash out (Figure 4C). The holding current significantly increased from −143 ± 19.9 pA to −269 ± 24.6 pA (*p* < 0.0001, *n* = 14) during nicotine application and the resting membrane potential (measured during a brief switch into current clamp mode) shifted to less negative values by an average of 7.07 ± 0.89 mV (*p* < 0.0001, *n* = 14) to an average of 48.0 ± 1.98 mV.

**Figure 5:**
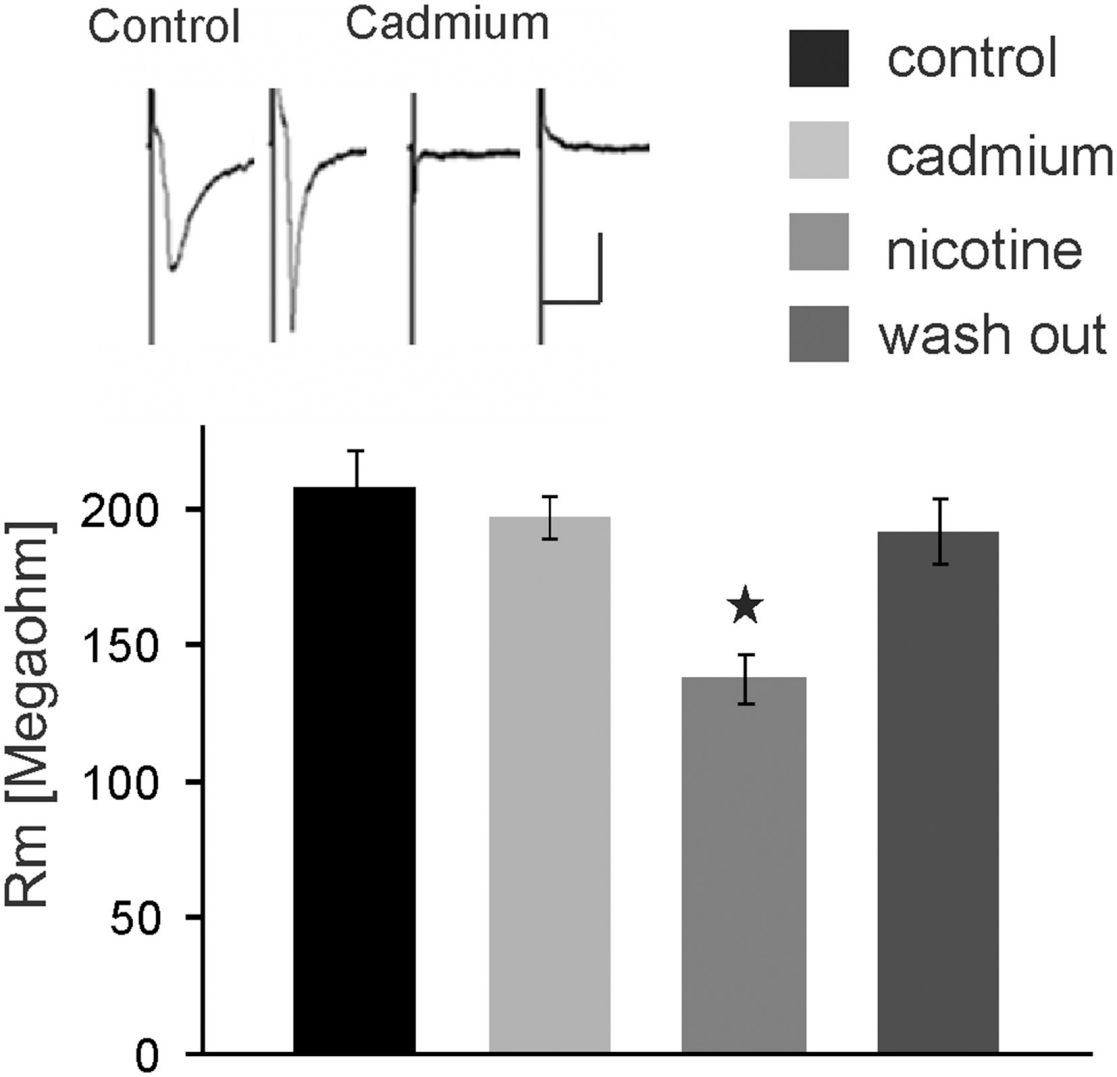
**The nicotine effect persists in presence of cadmium**. Auditory and trigeminally evoked EPSCs were completely blocked by bath application of 100 μM cadmium, see insert (horizontal bar indicates 25 ms and vertical bar100 pA.) The effect of nicotine on the membrane resistance in PnC giant neurons persists in presence of 100 μM cadmium, indicating that it does not require synaptic transmission. Asterisk marks statistical significance with *p* < 0.05 (*n* = 9 cells).

We further tested whether nicotine affects PnC giant neurons directly or through the activation of interneurons. Thus, we blocked voltage-gated calcium channels required for synaptic transmission by bath application of 100 μM cadmium. Auditory and trigeminal stimulation was used to verify the block of synaptic transmission. There was no significant effect of cadmium on the membrane resistance, as shown in Figure 5 (*p* = 0.75; *n* = 9). However, even during the blockage of synaptic transmission with cadmium, nicotine significantly reduced the membrane resistance of PnC giant neurons to 75 ± 5.9% of its original value to an average of 126 ± 10.47 MΩ (*p* = 0.03; *n* = 9), indicating that nicotine either activates receptors that are directly located on the recorded neurons, or it activates the presynaptic release of inhibitory neurotransmitter independently from the activation of voltage-gated calcium channels.

**Figure 6:**
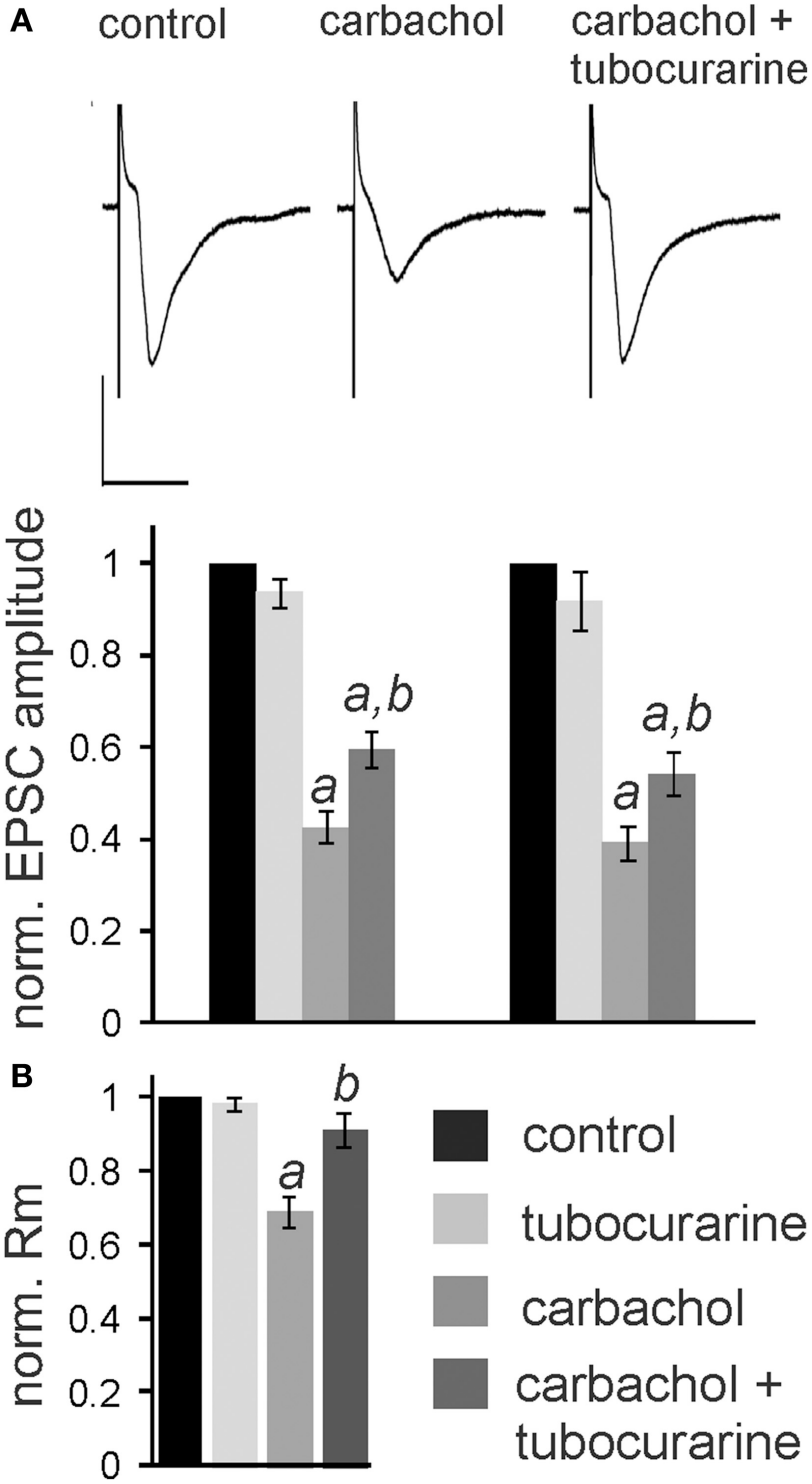
**Tubocurarine partly reverses the effect of carbachol on PnC giant neurons**. **(A)**, *top*: Exemplary traces of trigeminally evoked synaptic currents during perfusion of tubocurarine, carbachol, and the two drugs combined. Bars indicate 25 ms (horizontal) and 100 pA (vertical). *Bottom*: The perfusion of 50 μM tubocurarine had no effect on synaptic currents, whereas 10 μM carbachol significantly reduced synaptic current amplitudes. When tubocurarine application was combined with carbachol, the reduction of synaptic currents was significantly smaller than with carbachol alone (*n* = 10 cells for tubocurarine and tubocurarine plus carbachol, *n* = 14 cells for carbachol alone). Significant differences to control are labeled by “*a*,” significant differences to the carbachol condition are labeled by “*b*.” **(B)** Membrane resistance (Rm) normalized to the resistance of each cell under control condition. Tubocurarine had no effect on Rm, whereas the application of carbachol significantly decreased Rm. The additional application of tubocurarine completely reversed this effect (*n* = 10 cells).

In the following experiments we aimed to confirm that the observed nicotine effect is due to specific activation of nAChR in the PnC. We have previously shown that the non-specific cholinergic agonist carbachol inhibits PnC giant neurons and that this inhibition is only partly reversed by specific muscarinic blockers (Bosch and Schmid, 2006). We therefore tested, whether the specific nicotinic blocker tubocurarine reverses the inhibitory action of carbachol on PnC giant neurons. We added carbachol (10 μM) and tubocurarine (50 μM) to the bath solution. Tubocurarine is known to be effective specifically on neuronal nAChR at concentrations used in this study (Jensen et al., 2005). Carbachol significantly decreased trigeminally evoked EPSC amplitudes to 42.60 ± 3.84% of the control amplitude and auditory evoked EPSCs to 39.23 ± 4.02% of the control amplitude (*p* < 0.001 in both cases; *n* = 14; Figures 6A,B). Please note that the inhibition of EPSCs by carbachol was much stronger than the inhibition of EPSCs by nicotine shown above in Figure 3. Tubocurarine had no significant effect on EPSCs when given alone (*p* = 0.9 for both trigeminal and auditory stimulation; *n* = 10; Figure 5B). Perfusion of slices with tubocurarine, however, significantly reduced the inhibitory effect of carbachol. EPSC amplitudes were only reduced to 67.15 ± 4.86% of control amplitudes following trigeminal stimulation, and 56.19 ± 3.49% of control amplitudes following auditory stimulation (*p* < 0.001 in both cases; *n* = 10, Figure 6A). Furthermore, carbachol significantly decreased the membrane resistance to 69.58 ± 4.50% of control. The effect on membrane resistance was completely reversed by the additional application of tubocurarine [*F*_(2, 31)_ = 12.996; *p* = 0.001; Figure 6B]. *Post-hoc* Tukey adjustment revealed a significant difference between tubocurarine alone (98.91 ± 2.71% of control) and carbachol alone condition (*p* = 0.001); and also between carbachol and carbachol plus tubocurarine condition (*p* = 0.011). In summary, tubocurarine only partly reversed the carbachol effect on EPSC amplitude, while it completely blocked the carbachol effect on membrane resistance. This indicates that carbachol activates both muscarinic and nicotinic receptors, but that its effect on the membrane resistance of PnC giant neuron is exclusively mediated by nicotinic receptors.

**Figure 7:**
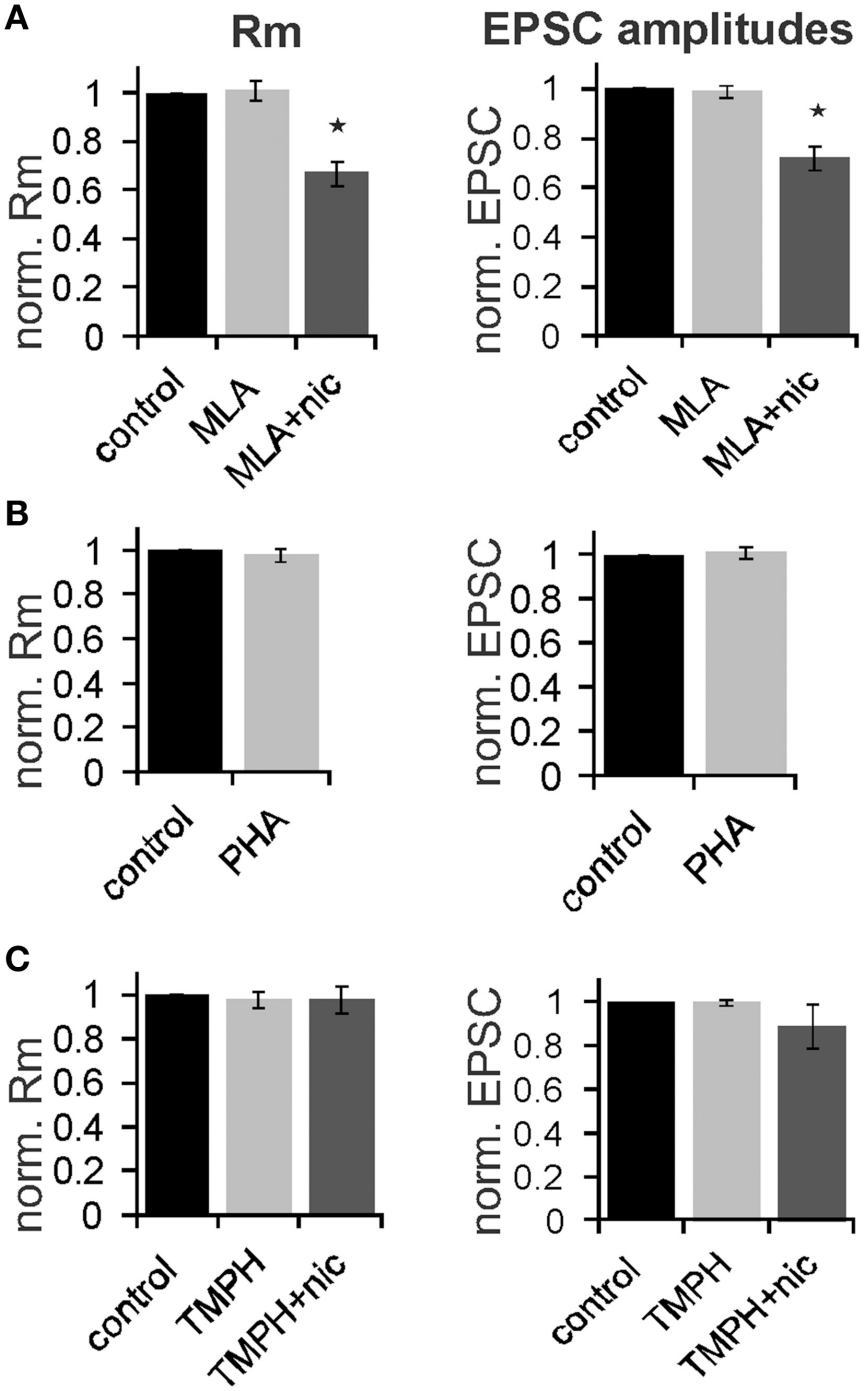
**The effect of nicotine on PnC giant neurons is mediated by non-α7-nicotine receptors**. **(A)** Effect of the α7nAChR preferring antagonist MLA (100 nM). Membrane resistance (Rm) was normalized to the resistance of each cell under control conditions. MLA alone had no effect on Rm, however, the effect of nicotine persisted in presence of MLA. MLA alone did also not affect EPSC amplitudes, whereas nicotine reduced synaptic currents in presence of MLA (*n* = 4 cells, only trigeminal EPSC amplitudes were analyzed). **(B)** Effect of the α7 specific agonist PHA543-613 (30 μM) on Rm and EPSC amplitudes: PHA 543-613 had no effect on PnC giant neurons (*n* = 6 and *n* = 4, respectively). **(C)** Effect of the non-α7 preferring antagonist TMPH (100 nM): TMPH alone had no effect on Rm or EPSC amplitudes. In presence of TMPH, nicotine did also not affect Rm and synaptic currents, indicating that TMPH antagonizes the nicotine effect. Asterisk marks statistical significance with *p* < 0.05 (*n* = 6 cells).

In order to determine the nAChR subtype responsible for the nicotine effect on PnC giant neurons we recorded under control conditions, and then added the α7nAChR preferring antagonist MLA (100 nM). After recording in presence of MLA, we added nicotine (10 μM) in order to see whether the nicotine effect is blocked in presence of the antagonist. There was a significant effect of drug on membrane resistance [*F*_(2, 18)_ = 4.45, *p* = 0.02; *n* = 4] and the *post-hoc* analysis revealed no effect of MLA alone (101 ± 3.9%; *p* = 0.13), and a significant effect of nicotine in the presence of 100 nM MLA (60 ± 5%; *p* = 0.001; Figure 7A). There was also a significant drug effect on EPSC amplitudes [*F*_(2, 18)_ = 4.5; *p* = 0.03; *n* = 4]. MLA alone did not change EPSCs (99.8 ± 2.7%; *p* = 0.13), but the nicotine effect persisted in presence of MLA, such that EPSC amplitudes decreased to 70 ±5% (*p* = 0.001; Figure 7A).

We also applied the highly specific α7 agonist PHA 543-613 (30 μm). The membrane resistance was unaffected by the perfusion of PHA with the resistance changing to 98 ± 2.1% from 192 to 189 MΩ (*p* = 0.69; *n* = 6). EPSC amplitudes were also unaffected by PHA (*p* = 0.21; *n* = 4; Figure 7B). We then tested the non-α7 preferring nAChR antagonist 2,2,6,6-Tetramethylpiperidin-4-yl heptanoate (TMPH hydrochloride, 100 nM) using the same experimental procedure as with MLA. We found no significant effect of drugs on the membrane resistance, indicating that pre-application of TMPH blocked the nicotine effect [*F*_(2, 16)_ = 2.41; *p* = 0.15; *n* = 6]. We also found no significant drug effect on EPSC amplitudes [*F*_(2, 16)_ = 2.31; *p* = 0.19; *n* = 6; Figure 7C].

In summary, our electrophysiological experiments support our behavioral results showing that the non-α7nAChR preferring antagonist TMPH blocks a portion of the cholinergic inhibition of startle neurons in the PnC, whereas the α7 antagonist MLA and the agonist PHA543-613 had no effect. Together with the behavioral results reported above our data indicate that non-α7nAChRs are expressed in the PnC and inhibit startle signaling, thereby contributing to PPI at 20–100 ms interstimulus intervals.

### Are non-α7nAChRs in the PnC responsible for enhancement of PPI through systemic nicotine?

**Figure 8:**
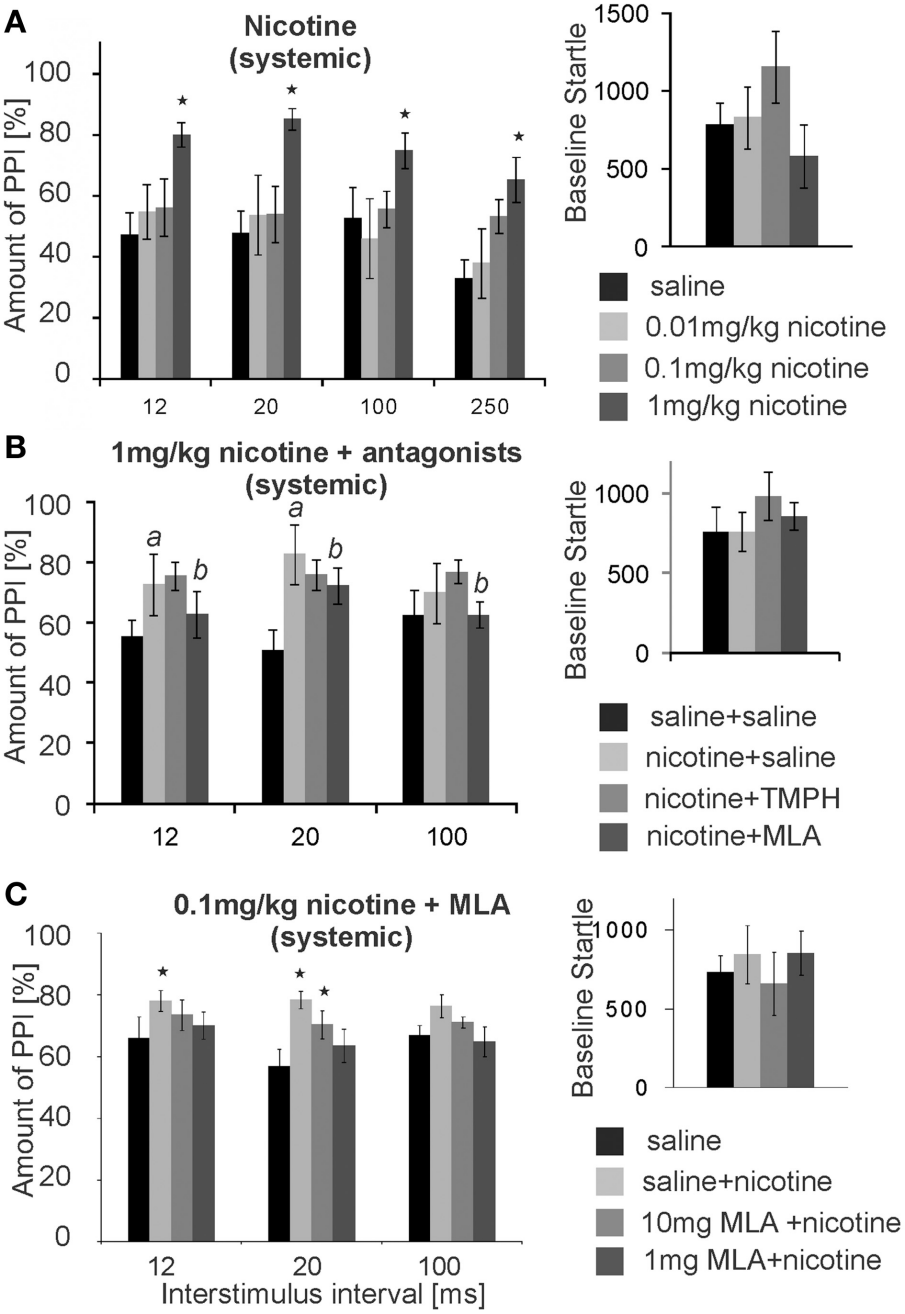
**Systemic nicotine enhances PPI, which can be reversed by systemic MLA, but not by systemic TMPH**. **(A)** Subcutaneous injections of nicotine significantly enhanced PPI of startle at the highest dose of 1 mg/kg, without changing the baseline startle amplitude (*n* = 8 rats). A low prepulse of 75 dB was used here. Nicotine did not significantly change baseline startle responses. **(B)** Another batch of rats injected with 1 mg/kg nicotine also showed nicotine enhanced PPI (significant difference between saline and nicotine condition labeled by “*a*”). An additional intraperitoneal injection of 5 mg/kg MLA, but not of 5 mg/kg TMPH or saline, significantly reduced the nicotine enhanced PPI (significant difference between nicotine and nicotine + MLA condition labeled by “*b*”). There was no drug effect on the baseline startle response (*n* = 8 animals). **(C)** With a shorter protocol and only one nicotine injection per rat, subcutaneous nicotine at the dose of 0.1 mg/kg significantly enhanced startle at short ISIs of 12 and 20 ms. This was completely reversed by 1 mg/kg MLA administered i.p., and partly reversed by 10 mg/kg MLA i.p. Asterisk marks statistical significance with *p* < 0.05 (*n* = 12 animals).

Numerous studies have shown that acute systemic nicotine enhances PPI. We confirmed this PPI enhancing effect in our laboratory, using a low intensity prepulse of 75 dB. Subcutaneous (s.c.) injections of nicotine significantly affected PPI [*F*_(3, 29)_ = 14.63; *p* < 0.0001; *n* = 8 animals; Figure 8A]. A LSD *post-hoc* analysis revealed that doses of 0.01 and 0.1 mg/kg had little effect, showing a significant increase in PPI only at an ISI of 250 ms with the higher dose. Systemic injections of 1 mg/kg nicotine (free base) significantly increased PPI from ~50 to 80% for ISIs of 12, 20, 100, and 250 ms. Baseline startle amplitudes were not significantly affected by s.c. nicotine, when compared to saline controls (*n* = 8; *p* = 0.25; Figure 8A). Testing of the higher dose of 10 mg/kg was abandoned, since it occasionally led to seizures.

After establishing the nicotine enhancement of PPI, we combined systemic nicotine injections with systemic injections of subtype-specific antagonists in a different batch of rats in order to see whether or not the non-α7nAChRs that contribute to PPI are also responsible for the nicotine enhanced PPI. A low prepulse of 75 dB was also used here to allow for PPI enhancement by nicotine. There was a significant effect of drug [*F*_(2, 21)_ = 50.27; *p* < 0.0001; *n* = 8; Figure 8B]. LSD *post-hoc* again confirmed a PPI enhancing effect of nicotine. Further, systemic MLA, but not TMPH, significantly attenuated nicotine enhanced PPI at ISIs of 12 and 20 ms. There were no significant effects of either TMPH or MLA on baseline startle amplitudes, in comparison to saline controls (*p* = 0.15).

In a different batch of rats we repeated the MLA injections using different concentrations. In order to avoid the possibility that nicotine receptor up- or down-regulation affects our results, each rat received only one dose of nicotine and a saline control injection (pseudorandomized). We also measured PPI at ISIs of 12, 20, 100 ms only, cutting down the total time of startle testing in order to avoid the possibility that the short-lasting nicotine effect was wearing off during testing. With a shorter protocol and a single dose per rat the effect of the lower dose of 0.1 mg/kg nicotine was also significant. Co-administration of 1 and 10 mg/kg MLA reversed the PPI enhancing effect of nicotine in a dose dependent manner (Figure 8C). ANOVA revealed an effect of drug [*F*_(3, 56)_ = 5.17; *p* = 0.003; *n* = 12] and *post-hoc* tests showed a significant effect of nicotine for the nicotine only measurements at 12 ms (*p* = 0.003) and 20 ms ISI (*p* = 0.04), but no significant change to saline control when MLA was co-administered, except from the less efficient MLA dose at 20 ms ISI (Figure 8C). Systemic TMPH tested at these conditions had still no effect on nicotine enhanced PPI [*F*_(3,56)_ =0.173; *p* = 0.916; *n* = 12; data not shown]. In summary, nicotine enhanced PPI seems to be dependent on α7nAChRs. Our previous *in vivo* and *in vitro* results indicated that nAChRs expressed in the PnC are of the non-α7nAChR type, since neither the α7nAChR agonist PHA 543-613 nor MLA applied to the PnC revealed any effect on PnC signaling or PPI (see above). In accordance with this, local microinfusions of MLA into the PnC did not affect nicotine enhanced PPI [*F*_(1, 23)_ = 2.21, *p* = 0.34; *n* = 12 animals; Supplementary Figure S3] or baseline startle (*p* = 0.81; *n* = 12; data not shown). In summary, systemic antagonism of α7nAChRs, but not local antagonism in the PnC, reversed the PPI enhancing effect of nicotine.

In conclusion, our present study indicates that nAChRs are functionally expressed in the PnC, which receives cholinergic input from the PPT. Both electrophysiological and behavioral data suggest that these are non-α7nAChRs and that they are activated during PPI, contributing to the inhibition of startle by prepulses. In contrast, the enhancement of PPI through systemic nicotine is mediated by α7nAChRs expressed elsewhere in the brain than in the PnC.

## Discussion

In order to identify the role of nAChRs in PPI, we employed systemic injections and stereotaxic microinfusions in combination with behavioral measurements and *in vitro* slice electrophysiology. Our studies suggest that non-α7nAChRs in the PnC contribute to startle inhibition by prepulses, whereas the PPI enhancing effect of systemic nicotine is mediated predominantly by α7nAChRs that are not located in the PnC.

### nAChRs in the PnC

Stereotaxic microinfusions into the PnC as well as our electrophysiological experiments clearly showed the expression of functional nAChRs in the PnC. The fact that local microinfusion of both nicotine and the nicotine antagonist TMPH into the PnC inhibited PPI seems puzzling at first glance; however, the presence of exogenous nicotine presumably makes these receptors unavailable for additional activation by acetylcholine released from PPT projections, therefore nicotine basically acts as an antagonist. Alternatively, the persistent presence of a high concentration of nicotine following PnC microinfusion could also simply inactivate nAChRs (Revah et al., 1991). We saw no sign of a decay of nicotine effect on PnC neuron excitability during the 10 min. nicotine bath application, which favors the first possibility. However, we would expect to see a reduction of baseline startle after local nicotine microinfusion due to reduced PnC excitability, which we did not find. Baseline startle amplitudes, though, can be very variable and e.g., potentiated by the microinfusion procedure itself, since it is mildly stressful and might lead to startle sensitization. We therefore, might have missed changes in baseline startle.

Our findings are in line with results of Stevens et al. (1993) who showed an antagonistic effect of tubocurarine on nicotine induced effects on postsynaptic currents of medial pontine reticular formation neurons. A previous study showed that activation of muscarinic receptors in the PnC inhibits synaptic transmission presumably through a presynaptic mechanism (Bosch and Schmid, 2006). We found an additional non-muscarinic receptor mediated effect of carbachol that affected the membrane resistance of PnC neurons (Bosch and Schmid, 2006, 2008). Our present data using carbachol in combination with tubocurarine further indicates that there is a nicotinic component contributing to the effect of carbachol on PnC neuron signaling, and that this nicotinic component is not affecting presynaptic glutamate release. Nicotine receptors are known to be cation channels that activate and inactivate rapidly. Neither our nicotine microinfusions, nor the bath application of nicotine are suitable to capture any of these rapid and short-lasting effects of nicotine receptor activation. Instead, we observe a tonic effect of startle-mediating PnC neurons through nicotine that reduces the membrane resistance and EPSC amplitudes while shifting the resting membrane potential toward less negative values. It has to be noted that since we didn't correct our data for the liquid junction potential, the measured resting membrane potential of −48 mV during nicotine application may correspond to a real membrane potential of around −60 mV. The observed nicotine effects are independent from the activation of cadmium sensitive voltage-gated calcium channels commonly required for neurotransmitter release. Also, it doesn't affect paired-pulse ratio of the glutamatergic EPSCs. Together, this suggests that nicotine does not affect presynaptic glutamate release probability. Future studies will have to show whether calcium influx through nAChRs directly activates presynaptic glycine or GABA release onto PnC giant neurons, and/or whether postsynaptic nAChRs affect PnC excitability through e.g., the activation of potassium, cation and/or chloride channels (for review see Shen and Yakel, 2009).

### Timing

Our present and past studies show the functional expression of both muscarinic and nicotinic receptors in the PnC, the sensorimotor interface of the startle circuitry. Microinfusion of nicotine and TMPH into the PnC in our study, as well as of muscarinic antagonists by Fendt and Koch (1999), significantly attenuated PPI, indicating that the activation of both muscarinic and nAChRs are likely contributing to PPI of startle. The generally limited effects of cholinergic antagonists indicate that the cholinergic inhibition might not be restricted to the PnC, but also affect sensory neurons upstream (Gómez-Nieto et al., 2014), and that other neurotransmitters, such as GABA, glutamate, and glycine, also contribute to PPI, as proposed before (Yeomans et al., 2010; Geis and Schmid, 2011).

Both ionotropic and metabotropic cholinergic and GABAergic receptors mediate PPI in rats (Jones and Shannon, 2000b; Yeomans et al., 2010, present data). Ionotropic receptors within the primary startle and PPI pathway would be expected to mediate PPI at rather short ISIs, due to their rapid activation and inactivation. In particular, α7nAChRs and GABA_A_ receptors have been shown to be most important for PPT at ISIs roughly from 20–100 ms (Figures 2, 3; Yeomans et al., 2010). Muscarinic receptors are slower to activate and yield longer lasting effects and should therefore, affect PPI at intermediate and longer ISIs. Indeed, it has been shown that systemic injections of GABA_B_ or muscarinic antagonists disrupt PPI at longer ISIs from 100–500 ms, but not at short ISIs (Jones and Shannon, 2000b; Fendt et al., 2001; Ukai et al., 2004; Yeomans et al., 2010), and PnC microinfusion of muscarinic antagonists as well as knock-out of muscarinic M4 receptors have been shown to disrupt PPI in rats at ISIs of 100 ms (Fendt and Koch, 1999; Koshimizu et al., 2012). This clearly corroborates the hypothesis that the serial activation of ionotropic and metabotropic receptors contribute to the fast, transient and the long lasting inhibition of startle by a prepulse, respectively.

### Source of acetylcholine

The mesopontine cholinergic projections from the pedunculopontine tegmentum (PPT) to the startle mediating giant neurons in the caudal pontine reticular nucleus (PnC) have been identified as a crucial structure for inhibiting startle during PPI (for review see Koch, 1999; Fendt et al., 2001). In general, the mesopontine cholinergic cell groups send mainly ascending projections to the collicullus, thalamus, and striatum promoting orienting and approach behavior, as well as eye saccades, while the descending projections seem to inhibit avoidance/escape responses (Fendt et al., 2001; Jones and Shannon, 2004; Mena-Segovia et al., 2008; Winn, 2008; Yeomans, 2012). Stimulation of the PPT has shown to increase acetylcholine release in the PnC (Lydic and Baghdoyan, 1993), however, based on our data we cannot assume that the only source of acetylcholine release in the PnC is the PPT projection. Lesions the adjacent laterodorsal tegmentum (LDT) has also been shown to disrupt PPI (Jones and Shannon, 2004), whereas in a recent study specific cholinergic lesions in the PPT almost completely abolished startle responses without necessarily impacting PPI (MacLaren et al., 2014). This indicates that either the LDT is directly involved in mediating PPI or that there is a more complex cholinergic regulation of PnC inhibition on the level of LDT/PPT (see Kohlmeier et al., 2012).

Interestingly, our finding of fast nicotinic receptor activation followed by slower muscarinic activation parallels results shown for other PPT/LDT outputs, e.g., to the colliculus as shown in monkeys and rats (Isa and Hall, 2009), or to the thalamo-cortical arousal system for improving sensory processing in cats and rats (Steriade, 1993).

### nAChR subtypes

Nicotine receptor modulators, especially of the α7nAChR subtype, are currently evaluated in many studies as a potential target for enhancing cognitive function (Young and Geyer, 2013; Young et al., 2013). Both major nAChR subtypes found in the mammalian brain, the α4β2 and/or α7, were reported to be involved in PPI or in other forms of sensory gating (for review see Adler et al., 1998; Schreiber et al., 2002; Leiser et al., 2009). Our electrophysiological and behavioral data indicate that the nAChRs in the PnC are mainly of the non-α7 subtype since the non- α7 preferring antagonist TMPH reversed nicotine effects, whereas the α7 specific antagonist MLA and agonist PHA 546613 had no effect. TMPH antagonizes the majority of subtypes found in non-α7 containing neural nAChRs, which include the α3, α4, β2, and β4 subunits (Damaj et al., 2005). The α4β2 nAChR subtype is the most common neuronal non-α7nAChR and it has been shown to be involved in cognitive processing (Changeux, 2010). It is therefore, the most likely candidate for mediating ionotropic cholinergic effects in the PnC, but further research is required to confirm this.

The involvement of non-α7nAChR is surprising, since the α7nAChR has been implicated in the past in PPI and cognitive function: Specific α7nAChR agonists have been shown to ameliorate sensory gating deficits (Adler et al., 1998; Stevens et al., 1998; Suemaru et al., 2004; Kohnomi et al., 2010), and α7nAChR knock-out mice show a mild PPI deficit in a recent study (Azzopardi et al., 2013), although previous studies had not reported any PPI deficits in these mice (Paylor et al., 1998; Young et al., 2011). However, our data indicate that non-α7nAChRs in the PnC contribute a small portion to normal PPI, whereas α7nAChRs elsewhere in the brain can exert a powerful modulation of PPI when activated e.g., by exogenous nicotine (see below).

### Systemic nicotine enhanced PPI

Previous studies have shown that systemic nicotine or nicotine agonists enhance PPI in rats (Acri et al., 1994; Curzon et al., 1994; Faraday et al., 1999; Schreiber et al., 2002). Our data extend this result by showing that a low dose of nicotine increased PPI at an ISI of 250 ms, whereas a higher dose of nicotine was more effective in enhancing PPI at shorter ISIs (between 12 and 50 ms), where maximum PPI is observed in rats. Both the timing of the systemic effect of nicotine, as well as the fact that systemic MLA, but not PnC microinfusions of any nicotine antagonists, reversed the systemic nicotine effect, indicate that systemic nicotine predominantly affects α7nAChR nAChRs in brain regions other than the PnC that extrinsically modulate PPI. Indeed, multiple brain areas that have been shown to modulate PPI (for review see Koch, 1999; Fendt et al., 2001), receive cholinergic input, and express α7nAChRs, which include the superior colliculus, thalamus, basal lateral amygdala, the substantia nigra (SN), the hippocampus, the striatum, and the medial prefrontal cortex (mPFC, Woolf, 1991). Most importantly, Azzopardi et al. (2013) has shown that α7nAChR knock-out mice do not show nicotine enhanced PPI, which is in accordance to our current pharmacological results in rats.

Almost all individuals with schizophrenia smoke cigarettes and show improvement in cognitive performance and normal PPI following nicotine consumption (Kumari et al., 1996; Forchuk et al., 1997; Adler et al., 1998; Postma et al., 2006). Our results indicate that this effect may be due to the activation of α7nAChRs. Interestingly, the α7 subunit of the nAChR has been identified as a susceptibility gene for schizophrenia (for review see Martin et al., 2004).

## Conclusion

In conclusion, the present study shows that activation of non-α7nAChRs in the PnC contributes a small, but significant portion to PPI at short interstimulus intervals, whereas activation of α7nAChRs elsewhere in the brain predominantly mediate the PPI enhancing effect of an acute dose of nicotine. This is an important piece in understanding not only the role of cholinergic neurotransmission in arousal, orienting responses and startle inhibition, but also for the correct interpretation of behavioral, preclinical, and clinical data as well as for developing drugs for the amelioration of PPI deficits and the enhancement of cognitive function.

### Conflict of interest statement

The authors declare that the research was conducted in the absence of any commercial or financial relationships that could be construed as a potential conflict of interest.
