# Supplementary material for: Nicotine receptors mediating sensorimotor gating and its enhancement by systemic nicotine
Source: Front Behav Neurosci. 2015 Feb 11;9:30. doi: 10.3389/fnbeh.2015.00030 (PMC4324144; doi:10.3389/fnbeh.2015.00030)
Supplement: Supplementary file 1 [file DataSheet1.PDF]

**Supplement**

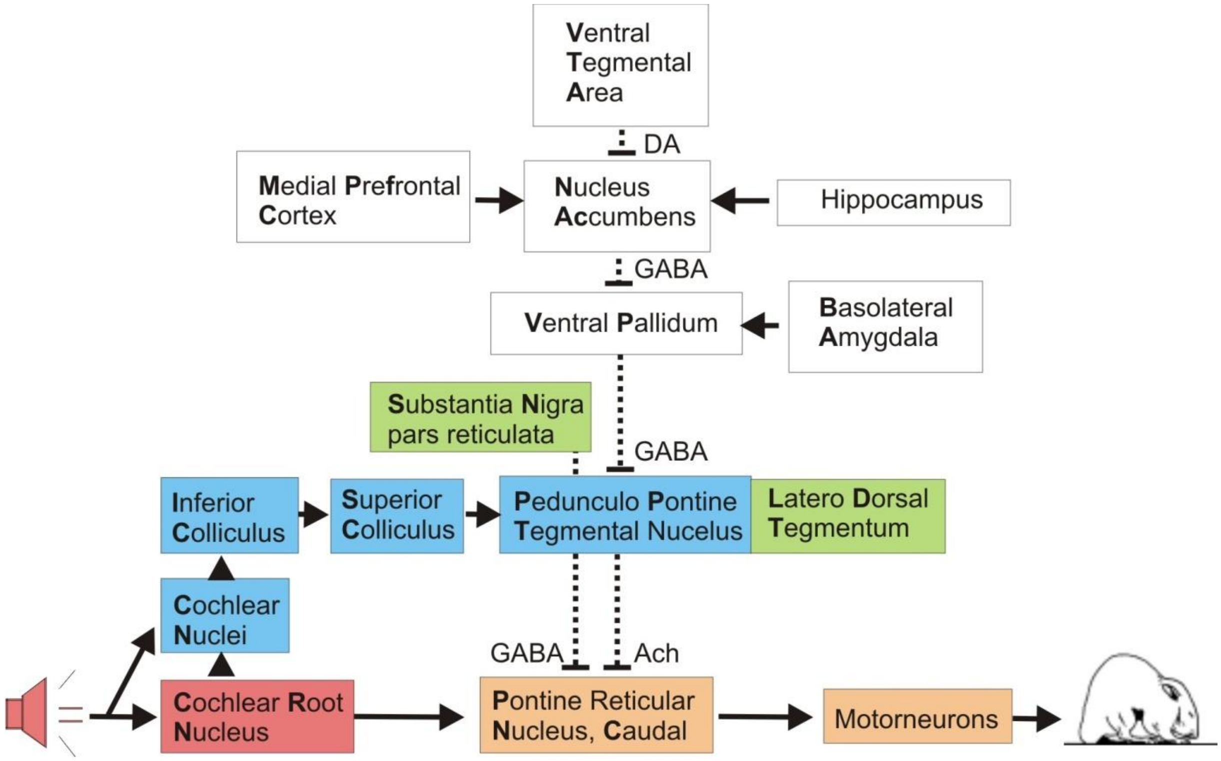


Fig. S1: Hypothetical pathways mediating the acoustic startle response and its modulation by prepulses. The red/orange pathway on the bottom represents the primary startle pathway. The blue pathway depicts the assumed pathway mediating prepulse inhibition as a fast forward inhibitory loop, with some ancillary structures in green. The structures in white are higher brain areas that have been shown to modulate startle responses and PPI (modified after Koch, 1999).

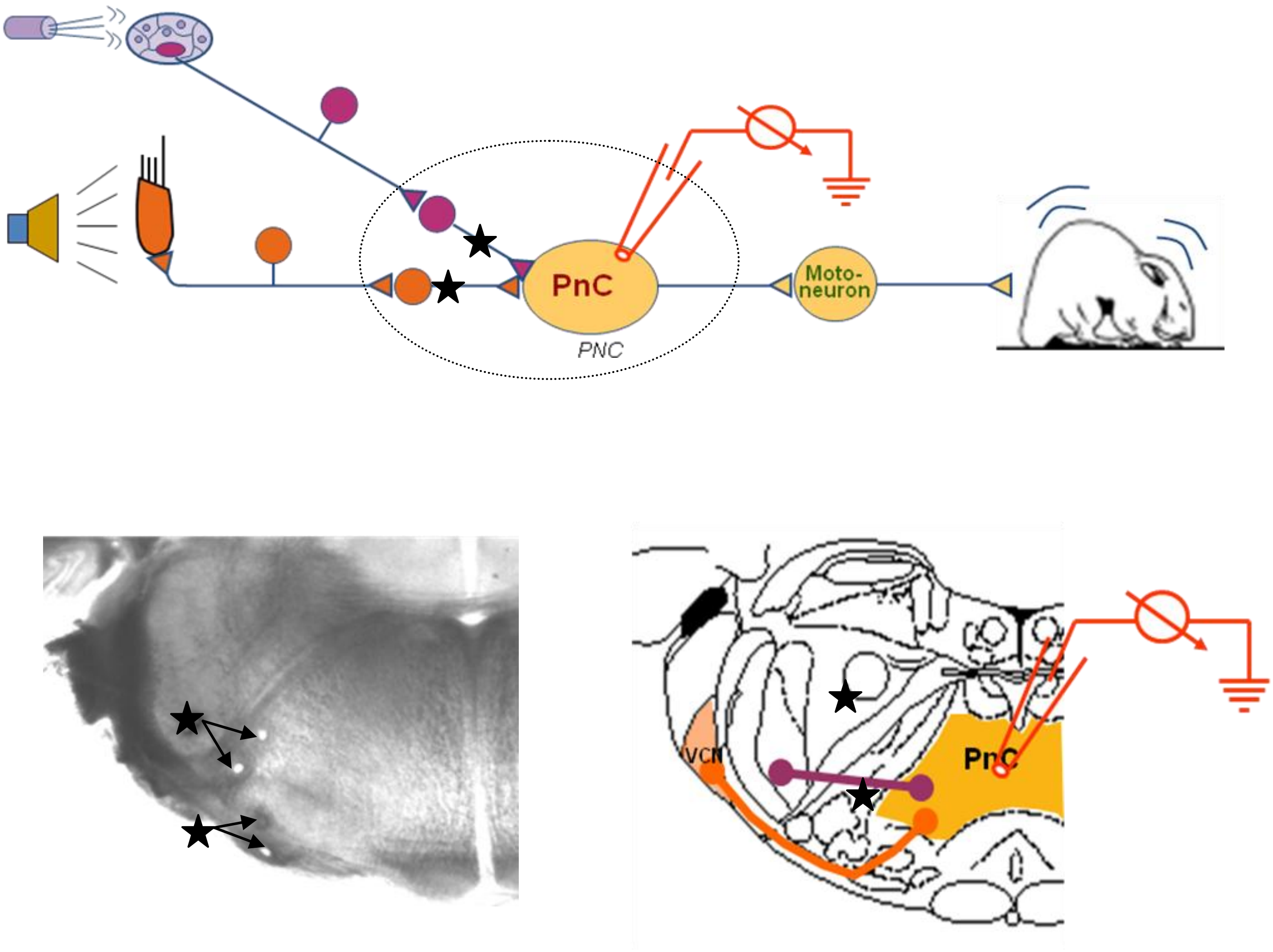


Fig. S2: Slice preparation containing the sensorimotor interface of the hypothetical auditory and trigeminal startle pathway. The dotted circle on the top shows the part of the startle pathway that is contained in the coronal 400 micrometer slice of the rat brain. Stimulation electrodes can be placed in order to specifically stimulate either the auditory or trigeminal afferent pathway (asterisks) to the PnC giant neurons. The PnC giant neurons can be visually identified by their soma size and localization within the PnC (see *bottom right*) for electrophysiological (patch-clamp) recordings. On the bottom left, a photo of a slice is shown with the paired holes where the bipolar stimulating electrodes had been placed in order to stimulate the afferent pathways (arrows).

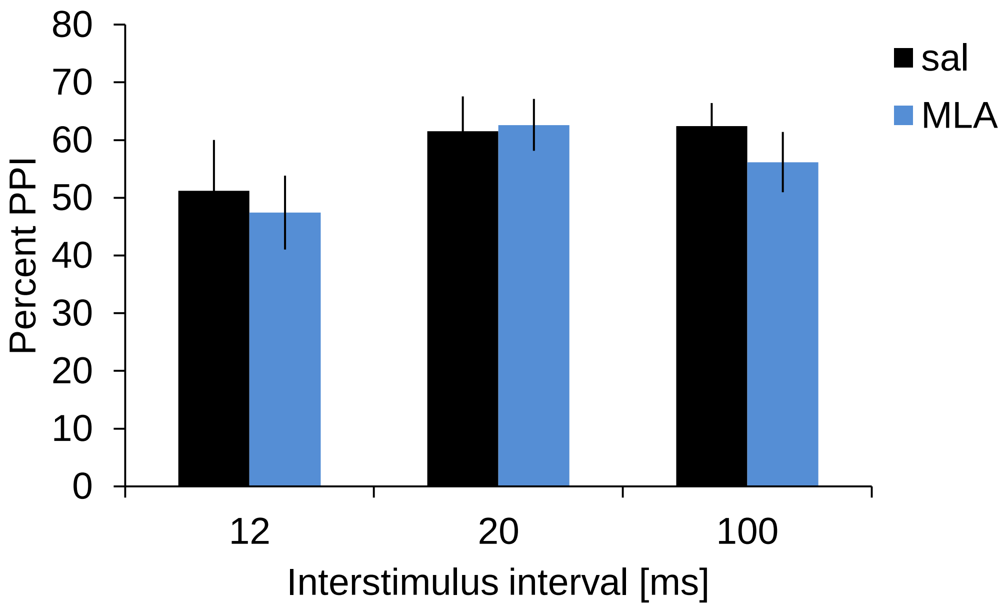


Fig. S3: Percent PPI after subcutanetous injection of nicotine combined with local microinfusions of either saline or MLA into the PnC. PPI was measured with a 75 dB prepulse at the insterstimulus intervals as indicated. Microinfusions of MLA into the PnC did not alter nicotine enhanced PPI (n=18 animals).
